# The neural networks of touch observation

**DOI:** 10.1162/imag_a_00065

**Published:** 2024-01-11

**Authors:** Michael Schaefer, Esther Kuehn, Felix Schweitzer, Markus Muehlhan

**Affiliations:** Department of Psychology, DNZE Tübingen, Tübingen, Germany; Hertie Institute for Clinical Brain Research, Tübingen, Germany; Institute for Cognitive Neurology and Dementia Research (IKND), Otto-von-Guericke University Magdeburg, Magdeburg, Germany; DZNE Tübingen, Tübingen, Germany; Department of Psychology, Faculty of Human Sciences, Medical School Hamburg, Hamburg, Germany; ICAN Institute for Cognitive and Affective Neuroscience, Medical School Hamburg, Hamburg, Germany

**Keywords:** social neuroscience, touch observation, fMRI, empathy, action observation

## Abstract

Studies have consistently demonstrated that the mere observation of touch engages our own somatosensory cortices. However, a systematic evaluation of the involved networks is missing. Here, we present results of a meta-analytic connectivity modeling (MACM) approach based on clusters revealed by activation likelihood estimation (ALE) combined with resting-state analysis to detect networks subserving our ability to empathize with tactile experiences of other people. ALE analysis revealed 8 clusters in frontal, temporal, and parietal brain areas, which behavioral domain profiles predominantly refer to cognition and perception. The MACM analysis further identified distinct networks that are subserved by subcortical structures, revealed that all clusters involved in touch observation are connected to dorso-medial frontal and anterior cingulate cortex control regions, and showed that medial temporal lobe memory structures do not inform network activation during touch observation (confirmed by post hoc resting-state connectivity analyses). Our data highlight the importance of higher-level control areas and suggest only a minor role for past bodily experiences in the ad hoc perception of other people’s experiences.

## 1 Introduction

The sense of touch is the first modality we develop early in life, and it plays an important role for interacting with the world (Gottlieb, 1971; Humphrey, 1964). The tactile sense is also one of the earliest senses that have emerged in evolution. For example, numerous single-celled organisms developed a tactile sense, whereas seeing and hearing evolved millions of years later only in multicellular animals (Hale, 2021; Kaas, 2008).

Since the famous stimulation studies by Wilder Penfield, we know that the postcentral gyrus is a major brain region to process and represent touch felt on our body (Penfield & Rasmussen, 1950). Brodmann area (BA) 1, BA 2, and in particular BA 3 receive thalamocortical projections from sensory fields and are described as the primary somatosensory cortex (SI) (Kaas, 2008; Viaene et al., 2011). Along with the secondary somatosensory cortex (SII), these brain areas are understood as crucial regions for processing tactile information in humans and animal species. Consequently, most studies that investigated human tactile experiences have focused on these early somatosensory processing areas. However, it is long known that somatosensory processing in the brain covers many more brain areas, including not only SI and SII but also the posterior insula, the precentral gyrus, the posterior parietal cortex, the temporal cortex, and other brain regions (Avanzini et al., 2016). It has been suggested that about 10% of all cortical surfaces may be linked to our tactile sense (Avanzini et al., 2016).

Given that touch processing activates extensive cortical territory, it is interesting to investigate the involvement of these networks when we observe other people being touched. Numerous fMRI studies have shown that the observation of touch on other people’s fingers, hands, necks, or legs activates the observer’s corresponding area representing the hand, neck, leg, or even single fingers in the SI or SII cortices (e.g., Blakemore et al., 2005; Ebisch et al., 2014; Keysers et al., 2004; Kuehn et al., 2013, 2014, 2018; Lee Masson et al., 2018, 2020; Schaefer et al., 2009; Schaefer et al., 2012b) but see Chan and Baker (2015). These mirror-like responses might also include the peripersonal space of the observed body (Schaefer et al., 2012a), but are stronger for actual observed touch compared to observing tactile stimuli merely approaching a body part (Kuehn et al., 2013). Also, studies employing Electroencephalography (EEG) or Transcranial Magnetic Stimulation (TMS) confirm these vicarious brain responses in the observer’s SI when seeing others being touched (e.g., Pisoni et al., 2018; Schirmer & McGlone, 2019). Based on this research, a role for somatosensation in perceiving and understanding social interactions has been suggested, which has been linked to simulation theories or embodiment resonance concepts (Keysers et al., 2010; Peled-Avron & Wolley, 2022). However, to the best of our knowledge, no meta-analytic study has yet been conducted to identify common networks activated during touch observation using available data sets and a data-driven analytical approach.

Most of the above cited empirical studies focus on “classical” somatosensory networks such as SI and SII (e.g., Blakemore et al., 2005), often even measuring brain activation in small portions of the cortex covering SI only (e.g., Kuehn et al., 2013, 2014, 2018). However, the processing of somatosensory information includes more complex computations involving additional brain networks, as pointed out above. For example, the posterior parietal cortex, but also motor networks are often reported to be engaged during touch observation (e.g., Meyer et al., 2011). With respect to motor networks, it is still not clear whether or not their involvement is mandatory during touch observation (Keysers et al., 2010). In addition, the anterior part of the insula has often been reported when observing touch, in particular linked to empathic feelings (Lamm et al., 2015). Furthermore, far too little attention has been paid to the potential involvement of subcortical structures and the medial temporal lobe including the hippocampus as well as higher-order control networks during touch observation. Subcortical structures play a particularly important role during actual touch perception, but dedicated studies that investigate if and in which cases also subcortical structures are activated during the observation of touch are so far lacking. This is particularly important given that those networks (and those situations) that trigger the involvement of subcortical structures may be particularly involved in learning by observation (Errante & Fogassi, 2020) and mental imagery (Hardwick et al., 2018) and may be more vulnerable to mixing up perceived from observed touch. The medial temporal lobe network on the other hand including the hippocampus may be particularly important for incorporating the observer’s own past experiences into a social interaction. For example, it has been shown that the action observation networks particularly activate when participants observe movements in which they themselves are trained compared to those which they have never performed before (Calvo-Merino et al., 2005, 2006; Galvez-Pol et al., 2020). Whereas this also involves procedural memory for motor actions that rely on non-hippocampal sources, for touch, an involvement of a ventral pathway including the somatosensory cortex, the insula, and memory-related networks, such as the hippocampal formation, could subserve the steady activation of sensorimotor networks during the observation of touches that encompasses own experiences (Bonda et al., 1996; Mishkin, 1979; Reed et al., 2004). One hypothesis is therefore that the hippocampus could contribute to these vicarious activations that are driven by the observer’s own experience. Finally, with respect to higher-order control areas, theory-of-mind has long assumed an important role of perspective taking and higher-order cognitive abilities for the emergence of empathy (Frith & Frith, 2005; Frith & Frith, 2012; Frith & Singer, 2008; Frith & Frith, 2003; Gallagher & Frith, 2003). However, for the case of touch, the importance of higher-order control areas is less described and therefore less clear.

The aim of the present study is to detect the neural networks underlying the observation of touch by specifically investigating those studies that measured whole-brain activation during touch observation to detect commonalities and differences between the different conditions and networks. To pursue this goal, we took a multi-step approach. First, we use an Activation Likelihood Estimation (ALE) meta-analytical approach to identify consistently activated cortical regions linked to seen touch. The ALE approach is based on a coordinate-based meta-analysis (e.g., Eickhoff et al., 2009) and allows us to answer the questions of which brain regions are commonly activated during touch observation across different research paradigms. Meta-analyses offer the advantage of being less biased to a specific study publication and/or experimental approach compared to investigating a single study sample, in particular when control mechanisms for publication bias are implemented (Acar et al., 2018). Second, the resulting clusters were characterized with respect to their behavioral significance and underlying paradigms using the BrainMap database. Finally, we employed a meta-analytic connectivity modeling (MACM) approach based on the clusters revealed by the ALE meta-analysis to determine the functional networks linked to touch observation. This allows us to investigate to which neuronal network nodes the above identified regions are commonly linked, and which networks are subserved by subcortical, memory, and higher-order control networks. These latter analyses were complemented post hoc by resting-state analyses to more specifically investigate the role of the medial temporal lobe in the network of touch observation. The reason for incorporating this additional analysis was that the finding of a missing involvement of the medial temporal lobe in touch observation (see below) was surprising, and we aimed at reducing the likelihood for reporting false negative results. Together, this study is the first that uses meta-analytic techniques to study the neuronal networks that underlie touch observation, which provides important information on underlying computations and cognitive mechanisms relevant for human social behavior.

## 2 Methods

### 2.1 Preregistration

The meta-analysis was preregistered on PROSPERO. The details can be found here: https://www.crd.york.ac.uk/prospero/display_record.php?RecordID=266316.

### 2.2 Literature search

For our analysis on neuroimaging studies that describe activation patterns of observed touch, we systematically examined electronic databases (PubMed, PsycINFO, Web of Science) until September 2021. The search strategy used the following search formula: touch [Title/Abstract] OR tactile [Title/Abstract] AND observation [Title/Abstract] OR sight [Title/Abstract] OR vision [Title/Abstract] AND (fMRI[Title/Abstract] OR functional magnetic resonance imaging [Title/Abstract].

We had no date limit for our searches. Studies were included if they were written in English language, peer-reviewed, compared observation of touch to animate or inanimate objects, used fMRI as imaging technique, and performed whole-brain voxel-wise data analysis. Thus, studies reporting effects based on a priori-defined ROIs or restricting analyses to a slab of the brain were not considered to prevent biases towards those regions. However, studies using functionally defined masks for further analyses were not discarded.

Our literature search resulted in 15 experiments (see Supplementary Fig. S1 for a flow diagram, based on PRISMA; Page et al., 2021). Supplementary Table S1 provides further information about the selected articles and their experimental designs. The identified studies were examined manually to extract peak voxel coordinates (foci) for each experiment. If necessary, the data were transformed into MNI coordinates. All coordinates were double-checked by a second researcher before entering the ALE-analysis. The final dataset included 14 studies with 15 experiments and a total of 230 participants. We included data of contrasts between observed touch to a human relative to observed non-touch, to an inanimate object, or to baseline.

The included studies used statistical thresholds between p < 0.001 (uncorrected) and p < 0.05 (corrected for multiple comparisons, FWE or FDR). All participants were healthy without any known psychiatric or neurologic histories. Supplementary Table S1 shows demographic data and details of the included studies.

### 2.3 Activation likelihood estimation

ALE is a coordinate-based method that tests for the greater than chance convergence (Eickhoff et al., 2009, 2012; Turkeltaub et al., 2012) of the activation coordinates between the different experiments (studies). First, the foci (coordinates) of the relevant contrasts of the suitable studies were extracted. Foci reported in Talairach space were transformed into MNI space using the “Lancaster” transformation (Laird et al., 2009; Lancaster et al., 2007) implemented in GingerALE v3.0.2 (http://brainmap.org). This was set as the default space for the present meta-analysis. The foci and the respective sample sizes of all suitable experiments and studies were then read into GingerALE as a foci file. A Gaussian three-dimensional probability distribution was modeled around the foci to account for the spatial uncertainty associated with imaging studies (e.g., between-subject and between-template variance) (Eickhoff et al., 2009). The higher the sample size of a study, the higher the weight that the foci of that study receive in the ALE. Modeling of all included foci results in a Modeled Activation Map (MAM). ALE is then used to compute the union of all study-specific MAMs to obtain the local convergence of the respective results. Finally, the significance of the ALE values is tested against random distributions using permutation tests. This allows us to test which regions show a significantly greater convergence of results than would be expected by chance. In this meta-analysis, 1000 permutations were applied. The analysis was performed at a voxel level of p ≤ 0.001, Family Wise Error cluster level corrected (cFWE) at p ≤ 0.05, as recommended for ALE meta-analyses (Eickhoff et al., 2016; Müller et al., 2018). The results are presented as an ALE map using Mango version 4.1 (http://rii.uthscsa.edu/mango/) on a standard MNI152 T1 brain template.

### 2.4 Behavioral characterization and paradigm analyses

To interpret the convergence clusters identified by ALE with respect to their significance for cognitive processes, the clusters were characterized using metadata from the BrainMap database. The advantage of this procedure is that researchers’ subjective biases in interpreting the cognitive functions of the clusters can be reduced, since we generated these interpretations by using reverse inferences from a data-driven approach, that is, database matching (Lancaster et al., 2012). The BrainMap database contains the results of more than 19800 functional experiments subdivided into five main behavioral domains (Action, Cognition, Emotion, Interoception, and Perception) and 60 subdomains. Since the BrainMap database uses Talairach space as the default, the ALE clusters were first converted to Talairach space using Mango version 4.1 software (http://rii.uthscsa.edu/mango/) and stored as binary regions-of-interests (ROIs). The analysis was performed using the “Behavioral Analysis Plugin” version 3.1 for Mango 4.1 (https://ric.uthscsa.edu/mango/plugin_behavioralanalysis.html). For each behavioral subdomain, this plugin tests the proportion of the database’s coordinates that fall within the ROIs and compares them to the proportion that would be expected if the coordinates were not clustered (i.e., freely distributed). The results are reported as Z-scores for each subdomain. Z-scores ≥ 3.0 are considered significant because they survived both the correction for the size of the ROI and a correction for the number of subdomains for multiple testing (Lancaster et al., 2012).

In addition to the behavioral analysis, a regional paradigm analysis was performed using the “Paradigm Analysis Plugin Version 1.6” for Mango v4.1. (https://ric.uthscsa.edu/mango/plugin_behanapara.html). Using metadata from the BrainMap database, we examined which of a total of 111 paradigm classes can be associated with the clusters identified in ALE. For these analyses, a Z-score of ≥3.3 was used as the threshold (https://ric.uthscsa.edu/mango/versionhistory.html#v401).

### 2.5 Meta-analytic connectivity modeling

A MACM approach was chosen to investigate the functional networks in which the ALE clusters are involved. MACM is a data-driven method for determining task-based functional networks based on co-variance of activations (Fox et al., 2014; Robinson et al., 2010). Thus, in conjunction with the BrainMap database, neuroimaging data from the last 20 years are included. Analysis was performed using BrainMap Sleuth v3.0.4 and BrainMap GingerALE v3.0.2 (http://brainmap.org). The ROIs that also served for behavioral domain analysis and paradigm analysis were used as seed regions to identify task-based networks using an ROI-to-whole brain approach. ROIs were uploaded to the image search function of Sleuth. In the Sleuth search, the following criteria were selected “Diagnosis: Normals,” “Context: Normal Mapping,” “Imaging Modality: fMRI,” and “Activations: Activations only.” The identified co-activation coordinates were exported as input data for GingerALE. MNI reference space was selected as the output format. Subsequently, ALE analyses were performed for all foci files as previously described. The same correction thresholds and permutation tests were chosen as in the main analysis (1000 permutations, cluster-forming threshold p ≤ 0.001, cluster-level threshold p ≤ 0.05 FWE corrected for multiple comparisons).

### 2.6 Estimation of publication bias (file drawer problem) and the potential influence of dominant studies

To estimate the robustness of ALE clusters to potentially unpublished studies (file drawer problem), we used a fail-save N (FSN) calculation adapted for ALE as described by Acar et al. (2018).

The FSN is defined as the number of noise studies that can be added to the meta-analysis before the results change. In addition, a “lower boundary” was defined above which clusters are considered robust. This boundary applies equally to all ALE clusters and is determined by the studies included in the meta-analysis and the assumed publication bias. In a recent study, publication bias in neuroimaging studies was estimated to be between 6% and 30% (Samartsidis et al., 2020). For a conservative analysis, we therefore assume that there are 30% unpublished null findings, which in our case corresponds to five studies (30% of 15 studies). These null findings are simulated by noise studies that are similar to the original studies in terms of sample size and number of foci, but whose foci are randomly distributed across the brain. The noise studies were created using RStudio (version 4.1.2) and the algorithm provided by Acar et al.(2018). The noise studies were then integrated into the dataset and the meta-analysis was recalculated. If the results remain significant, the number of noise studies was successively increased until the cluster in question was no longer significant, at which point the FSN is reached. FSN values below the lower boundary indicate low robustness against publication bias.

Another issue is that ALE results may be influenced by a few dominant studies with large sample sizes (Acar et al., 2018). To estimate this effect, we set an individual “upper boundary” for each cluster based on the number of studies contributing to that cluster (Table 1). According to Acar et al. (2018), the number of null studies for the upper bound was set such that at least 10% of the original studies must still contribute to the effect of a cluster. The calculation was done according to the following formula: ((number of studies contributing to a cluster)/0.1)-(number of studies included in the ALE meta-analysis). FSN values above the upper limit mean that the results are driven by a few dominant studies. In the best case, FSN values are between the lower and upper bounds. The ALE-adjusted FSN calculation thus supports an appropriate interpretation of the ALE results.

**Table 1.** ALE clusters significant after cluster-level FWE correction for multiple comparisons.

Cluster #	Anatomical label^a^	Peak voxel coordinates (MNI)			BA	ALE^b^	Cluster size (mm³)	Center of mass (x, y, z)	No. of contributing experiments (%)	FSN
		x	y	z						
1	Postcentral Gyrus	-58	-22	38	2	0.0204	3880	-57.6, -25.3, 26.2	9	68
	Postcentral Gyrus	-58	-20	20						
	Superior Temporal Gyrus	-62	-42	20						
	Insula	-56	-38	22						
2	Parietal Lobe, Sub-Gyral	-30	-42	54		0.0172				
	Superior Parietal Lobule	-32	-52	56	7		3560	-33.4, -45.9, 52.2	10	83
	Inferior Parietal Lobule	-38	-40	50						
3	Middle Frontal Gyrus	42	6	40	6	0.0192	1792	45.8, 5.1, 38.5	6	41
	Precentral Gyrus	50	6	34						
4	Inferior Temporal Gyrus	-46	-72	2		0.0199	1776	-47.2, -70.2, 0.8	6	42
5	Temporal Lobe, Sub-Gyral	50	-60	-2		0.0171	1408	48.6, -63.3, 1.1	6	34
	Middle Temporal Gyrus	48	-58	10						
	Inferior Occipital Gyrus	46	-74	-2						
6	Precentral Gyrus	-56	2	36		0.0215	1224	-54.8, 2.2, 37.1	5	<30
7	Postcentral Gyrus	64	-18	30			1064	63.3, -16.2, 29.2	5	<30
	Precentral Gyrus	62	-14	40	4	0.0176				
	Postcentral Gyrus	62	-16	18						
8	Declive (Cerebellum)	20	-82	-10		0.015	720	16.2, -84.8, -7.4	2	<30
	Lingual Gyrus	12	-88	-6	18					

a Anatomical labeling according to Talairach Daemon (nearest gray matter within 5 mm, talairach.org) associated with the peak coordinates after icbm2tal transformation (GingerALE output).

b Maximum ALE value observed in the cluster.

BA, brodman area; L, left hemisphere; R, right hemisphere; x, y, z coordinates provided in MNI space.

### 2.7 Resting-state analysis

Contrary to our expectation, MACM analyses did not reveal co-activations in hippocampal regions (see Results). Therefore, as a post hoc analysis, we investigated the connectivity of the ALE clusters with the hippocampi by performing additional task-independent resting-state analyses.

The fMRI data used for the resting-state analysis originate from a previous project (Muehlhan et al., 2020). Eighty-eight participants (females: N = 41, age: 24.2 ± 2.95 years; males: N = 47, age: 24.47 ± 2.55 years) were scanned with a Siemens Trio Tim 3 T MR Tomograph using a 12-channel head coil. Structural images were obtained by using a Magnetization Prepared Rapid Gradient Echo Imaging (MPRAGE) sequence (repetition time (TR) 1900 ms, echo time(TE) 2.26 ms, flip angle α = 9°). Functional data were acquired using an echoplanar imaging (EPI) sequence with the following parameters: 200 whole-brain scans with 38 axial slices in ascending order; voxel size of 3.4 mm x 3.4 mm x 3.0 mm, 25% gap; TR 2200 ms, TE 25 ms, and flip angle α = 80°. Each slice had a matrix size of 64 × 64 voxels, resulting in a field of view of 220 mm. The total duration of the resting-state scan was 7.33 minutes.

Data were preprocessed within the standard processing pipeline of the CONN toolbox v 21a CONN-toolbox v 21a (Whitfield-Gabrieli & Nieto-Castanon, 2012) running in Matlab 20 b (mathworks.com) with the following processing steps: spatial realign and unwarp, slice timing correction, segmentation, direct normalization of the individual structural and functional images to their respective MNI templates, and spatial smoothing with an 8 mm kernel. These preprocessing steps are based on SPM12 v7771 (Wellcome Department of Imaging Neuroscience, UCL, London, UK). In addition, data artefacts related to movements greater than 0.5 mm to the previous frame or changes in global mean intensity greater than 3 standard deviations (SD) from the mean image intensity for the entire scan were detected prior to the functional normalization using the implemented Artifact Detection Tools (www.nitrc.org/projects/artifact_detect). Subsequently, a denoising procedure with a bandpass filter of 0.008 Hz – 0.09 Hz and a component-based correction (Behzadi et al., 2007) was applied to remove motion artifacts, physiological and other artifactual effects from the fMRI-signal. The time series characterizing the estimated participant motion (three rotation and three translation parameters and six other parameters representing their first-order time derivatives), the outliers detected by the Artifact Detection Tools, and the time series within the individual white matter and cerebrospinal fluid masks were included as first-level nuisance covariates and removed from the functional data.

The clusters identified in the ALE analysis (C1–C8, Table 1) were stored individually as binary masks and used as ROIs for the analysis of task-independent connectivity in resting state. Because the hippocampus has different connectivity patterns along the longitudinal axis (Adnan et al., 2016; Vogel et al., 2020), separate ROIs were created for the rostral and caudal hippocampal subregions using the Brainnetome Atlas (http://brainnetome.org/). Subsequently, ROI-to-ROI (RRC) analyses were performed using the CONN-toolbox v 21a based on SPM 12 v 7771 (Wellcome Department of Imaging Neuroscience, UCL, London, UK) running in Matlab 20 b. At the first statistical level, the Fisher-transformed bivariate correlation coefficient between the averaged BOLD time series of each pair of ROIs was calculated. At the second level, a general linear model (GLM) was applied to calculate the effect of interest, in our case the average deviation of connectivity from zero between each pair of ROIs, resulting in a single connectivity matrix of T-values. Thus, CONN-default settings for cluster-based inference were applied (pconnection ≤ 0.05, pFDR cluster level corrected at ≤ 0.05).

## 3 Results

### 3.1 ALE results

**Fig. 1.**
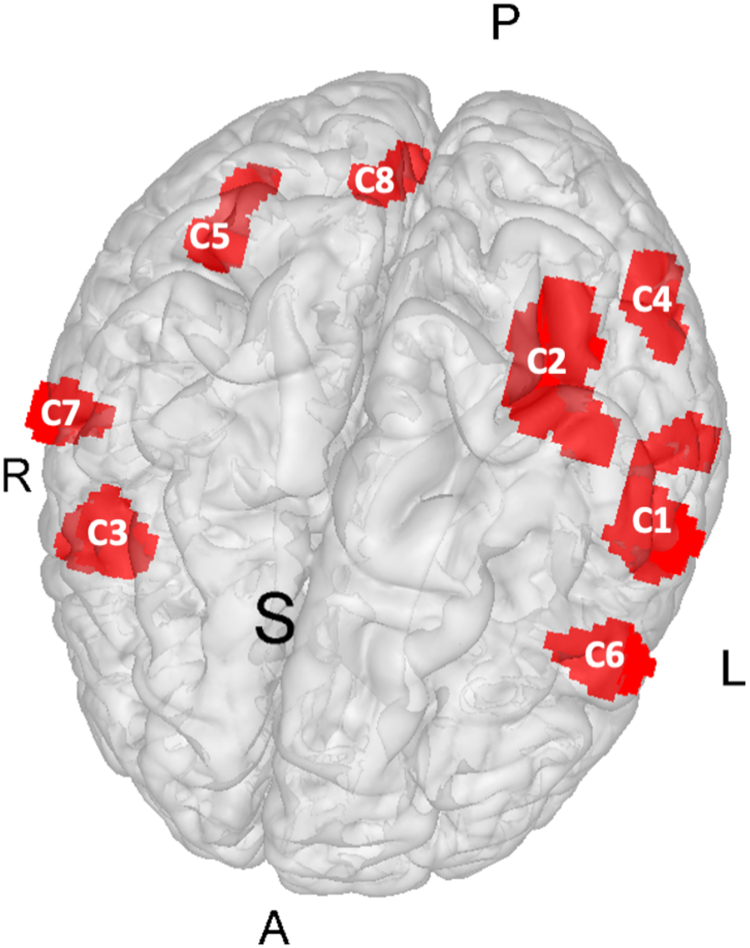
Results of the ALE meta-analysis overlaid on a gray matter glass brain (MNI). The 8 clusters represent significant convergence of touch observation relative to control conditions. Detailed coordinates can be found in Table 1. A: anterior, L: left, P: posterior, R: right, S: superior, This image was created with Mango v4.1. (http://ric.uthscsa.edu/mango/).

The ALE meta-analysis was used to detect greater than chance convergence of the activation coordinates between the included studies. The ALE meta-analysis revealed 8 clusters of convergence here referred to as C1 to C8, ordered by size (see Fig. 1 and Supplementary Figs. S2 and S3). Cluster C1 was mainly located within the left parietal lobe. 49.9% of C1 covers the postcentral gyrus, 20.2% the inferior parietal lobule, 19.2% the superior temporal gyrus, 8% the posterior insula, and 2.1% the transverse temporal gyrus. C2 was also located within the parietal lobe slightly superior to C1. 36.4% of C2 covers the Inferior parietal lobule, 29.9% the superior parietal lobule, 21.8% are located sub-gyral, 9.2% are located within the precuneus, and 2.7% covers the supramarginal gyrus. C3 covers parts of the right frontal lobe. 49.1% of C3 lies in the precentral gyrus, 39.3% in the middle frontal gyrus, 7.1% in the inferior frontal gyrus, and 4.5% are located sub-gyral. C4 is a left posterior cluster in which 30.2% are located in the middle occipital gyrus, 30.2% in the inferior temporal gyrus, 18.9% in the middle temporal gyrus, 14.4% are sub-gyral, and 6.3% covers the inferior occipital gyrus. C5 was also located within the occipital and temporal lobes but in the right hemisphere. 45.5% of the cluster is classified as sub-gyral, 33.5% is related to the middle temporal gyrus, 14.2% to the inferior occipital gyrus, 4% to the middle occipital gyrus, and 2.8% to the inferior temporal gyrus. From cluster C6, 97.4% cover the left precentral gyrus, and 2.6% the left middle frontal gyrus. C7 was mainly located within the right parietal gyrus. 82.7% covers the postcentral gyrus, 9% the inferior parietal lobule, and 8.3% the precentral gyrus. The smallest cluster C8 covers 73.3% of the right lingual gyrus, and 25.6% of the right cerebellar declive. Peak coordinates, cluster sizes, BAs and ALE values are summarized in Table 1.

### 3.2 Behavioral characterization and paradigm analyses

**Fig. 2.**
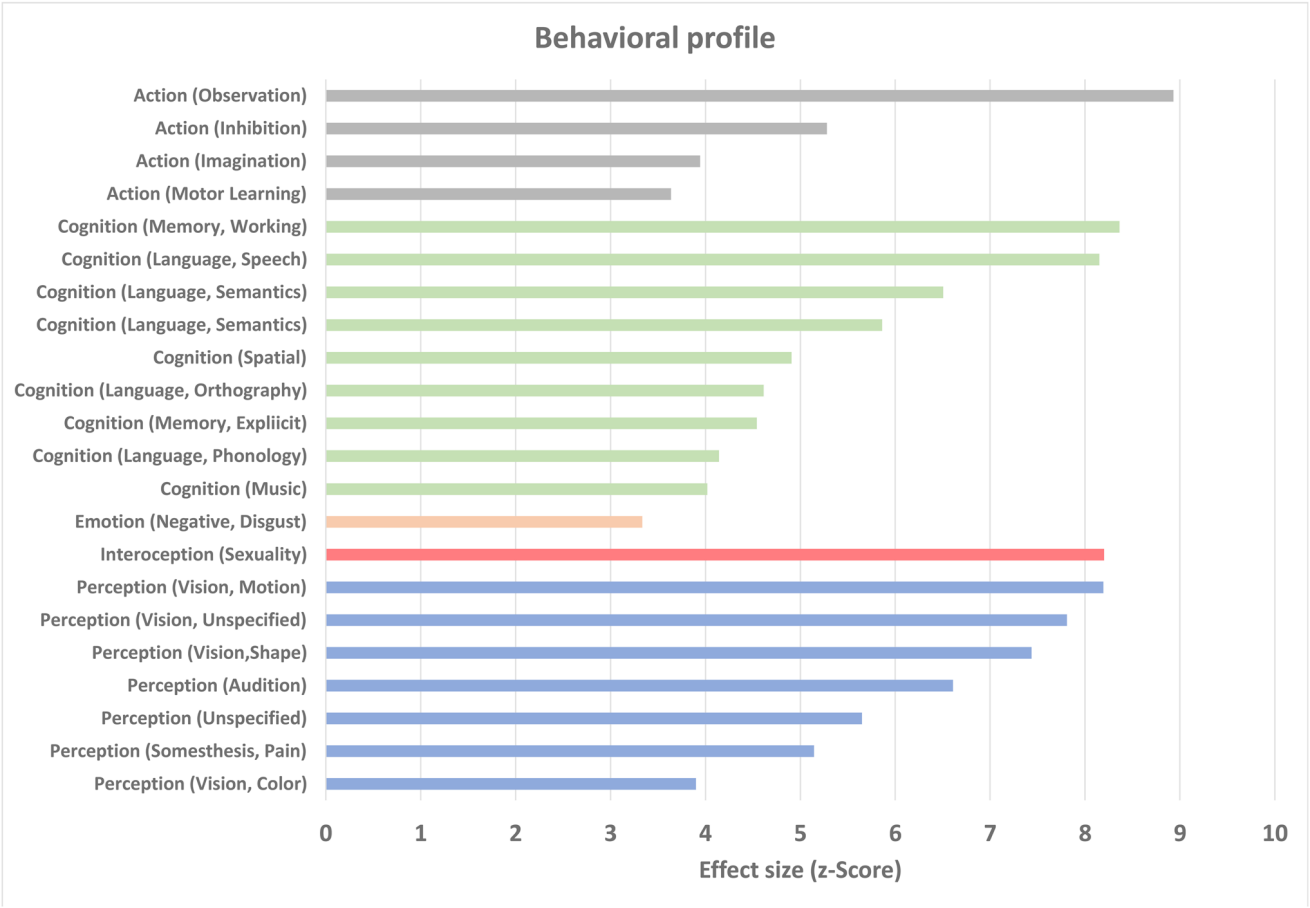
Behavioral characterization of ALE clusters. Behavioral domain profile shows that the network can be linked in particular to domains of cognition and perception. Only domains with an effect size of Z > 3 are shown, as these remain significant after correction for ROI/mask size and number of subdomains.

To investigate their associated cognitive processes, clusters C1–C8 were characterized using metadata from the BrainMap database. The behavioral domain profile indicates that the network is connected to different subdomains. The most represented domain is the domain “Cognition.” More precisely, the results of the subdomain analyses indicate that the network is associated with processes of “Working Memory,” “Language,” “Spatial Cognition,” “Explicit Memory,” and “Music Cognition.” The second largest domain is the domain “Perception” with a particular involvement of the visual subdomains “Motion,” “Shape,” “Color,” and “Unspecified.” The latter describes a category for all functions that cannot be directly assigned to the other (visual) subdomains. The domain with the strongest z-score is the domain “action observation.” The action domain also shows a particular involvement of the subdomains “Inhibition,” “Imagination,” and “Motor Learning.” Furthermore, the identified clusters were also associated with the subdomains “Interoception: Sexuality and Emotion: Disgust.” An exact description of the different subdomains can be found here: https://brainmap.org/taxonomy/behaviors/. The z-scores of each identified subdomain are depicted in Figure 2.

The paradigm analysis revealed a total of 33 paradigms that are related to the identified ALE-clusters C1–C8. These are summarized graphically in Supplementary Figure S4. A detailed description of all paradigm classes can be found here: https://brainmap.org/taxonomy/paradigms/. The results of the separate analyses for each of the 8 clusters can be found in Supplementary Table S2.

### 3.3 MACM results

**Fig. 3.**
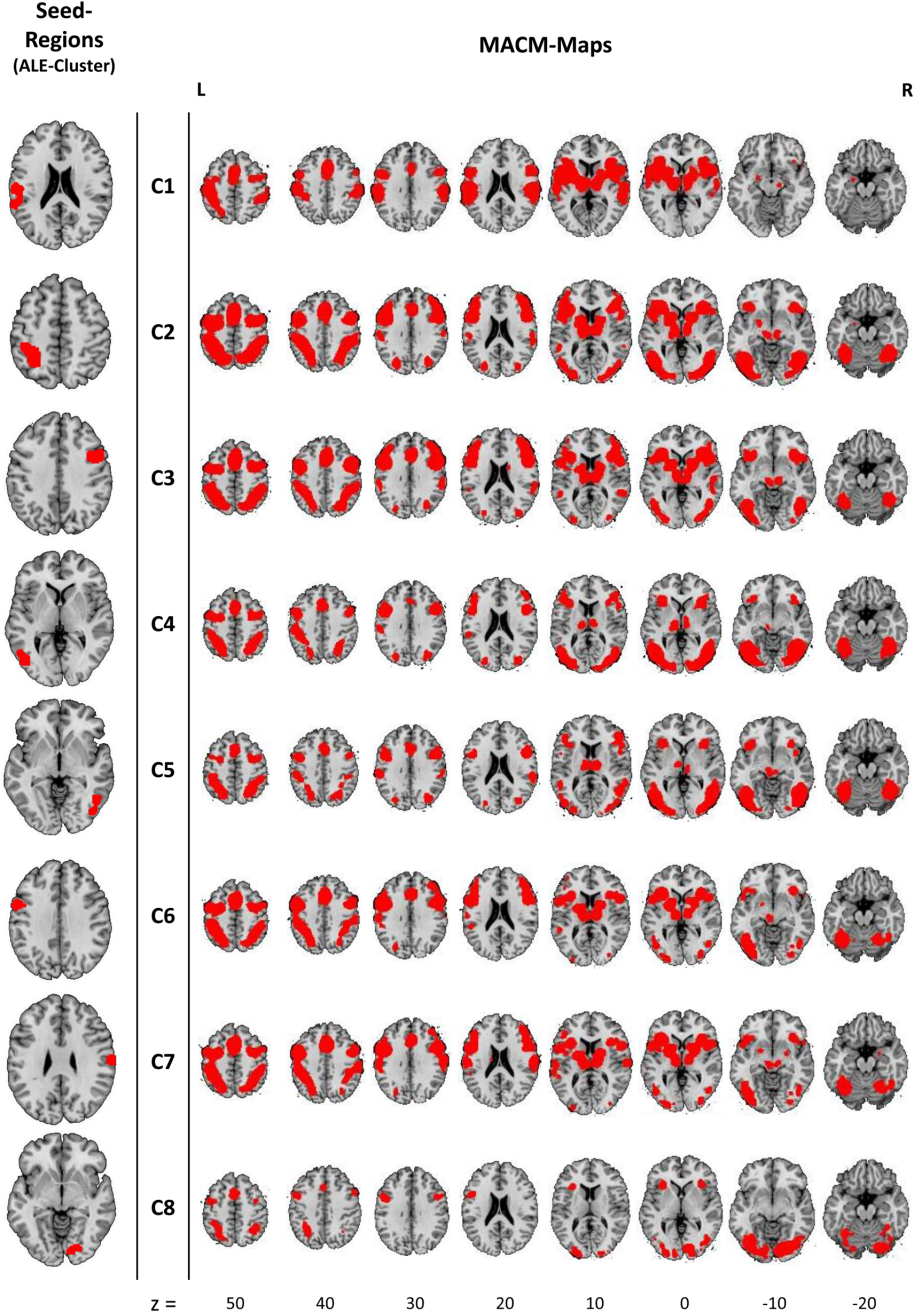
Results of the meta-analytic connectivity modeling (MACM). Shown are the seed regions of the 8 clusters on the left and the corresponding MACM maps on the right. All clusters are significant at a cluster-forming threshold of p < 0.001 and FWE cluster level corrected at p < 0.05. Z values indicate axial slice position in MNI space. This image was created with Mango v4.1. (http://ric.uthscsa.edu/mango/).

MACM was then applied to investigate the functional networks in which the 8 ALE clusters are involved. The MACM analyses of the 8 clusters revealed extensive networks associated with the identified clusters (Fig. 3 and Supplementary Material S6 (html-files for a three-dimensional representation with labels of the MACM networks)). It is worth highlighting that there is an almost complete overlap of all identified MACM networks, suggesting that each of the clusters identified with the ALE analysis relates to the same, or a very similar, neural network (Supplementary Video S7 a-c). In addition to the brain areas identified with the ALE-main analysis (see above), the MACMs revealed an association of the basal and thalamic nuclei as well as the dorsal anterior cingulate cortices and the medial and superior frontal cortices, as well as the regions of the ventral visual pathway. All peak voxels and labels are listed in Supplementary Tables S3. Furthermore, the task-independent resting state analysis revealed that the connectivity to the hippocampus is low or absent for all ALE clusters (Supplementary Fig. S5).

### 3.4 Fail-safe-N calculation

The FSN for ALE-clusters C1–C6 is between the upper boundary and the lower boundary, indicating that these clusters are robust to noise studies (i.e., potential publication bias) and are not driven by a few dominant experiments (with large sample sizes). The FSN for clusters C7- and C8 is below the lower boundary and is thus not considered robust against publication bias (Acar et al., 2018). The exact FSN and number of contributing studies are provided in Table 1.

## 4 Discussion

We here combined meta-analytic approaches with empirical resting-state functional connectivity analyses to study the brain networks subserving the observation of touch. In prior research, analyses have often focused on overlapping activity patterns between perceived touch and observed touch, and/or have investigated small portions of the brain in a limited number of participants only. Therefore, so far, the whole-brain neuronal networks that underlie our ability to show “empathy for touch” remain poorly described. ALE analyses revealed 8 clusters in frontal, temporal, and parietal brain regions that show consistent activation across studies in response to touch observation. Behavioral domain analyses link these clusters predominantly to cognition and perception. Additional MACM analyses demonstrate that all clusters correspond to the same neural network, characterized by an engagement of dorsomedial frontal and dorsal anterior cingulate cortices for all clusters. This points towards a contributing role of higher-level control brain areas during the observation of touch. Interestingly, we do not find an engagement of medial temporal lobe structures including the hippocampus neither for the observation of touch nor for networks subserving this function. This was also confirmed using empirical resting-state functional connectivity analyses. Given these areas are known to be involved in episodic memory formation, we suggest that the neuronal networks activated during touch observation are not directly subserved by the episodic remembering of tactile experiences on one’s own body but might be triggered by processes of perspective-taking and/or procedural memory mechanisms that rely on non-hippocampal sources.

Is the network that we describe here unique for the observation of touch or is it part of a broader network described previously for action observation? There are several studies using an ALE meta-analytic approach to examine the neural correlates of action observation (or imitation) (e.g., Caspers et al., 2010; Hardwick et al., 2018; Molenberghs et al., 2012; Papitto et al., 2020; Watson et al., 2013; Yang & Hofmann, 2016). Capsers et al. reported a bilateral network including premotor, SI, inferior parietal, intraparietal, and temporo-occipital brain areas, with a pronounced engagement of the lateral premotor and parietal cortex when observing or imitating actions. These results were taken as an argument for the existence of a widespread network representing action observation, which does not only include ventral premotor and inferior parietal cortices, as suggested in the traditional understanding of mirror neuron brain areas. The neural network of touch observation as described here revealed similar brain regions in frontal, temporal, and parietal brain regions. In addition, behavioral characterization revealed that the identified network is related to many domains relevant to touch observation, such as action observation and imagination, spatial cognition and working memory, and visual perception of motion, shape, and color. Using paradigm analysis, we linked this network to specific tasks that may involve parts of touch observation, for example, “film viewing,” or sub-components of touch observation, for example, “visuospatial attention,” “visual object identification,” to name just a few examples with particularly high effect sizes. In our view, this confirms our interpretation of an integrated somatosensory-motor network that supports not only touch observation but also film viewing and visual object identification. Recent studies are in line with this view, showing, for example, that neuromodulation (perturbation) of SI results in altered activity in known nodes of the action observation network (Valchev et al., 2016). The view of a role of SI for action observation is also supported by lesion studies (Binder et al., 2017; Bonivento et al., 2014; de Wit & Buxbaum, 2017). In this view, both touch observation and action observation may be represented by an integrated somatosensory-motor network (including in particular somatosensory cortices, premotor cortex, and inferior parietal lobe) (e.g., Keysers et al., 2018).

What may be the specific role of the somatosensory cortices in this somatosensory-motor network? Somatosensory activation during touch observation may be classified as part of a bottom-up process that is relevant to link other’s sensory experiences to one’s own sensory experience. It has been suggested that somatosensory brain regions may help to consciously feel sensations and action intentions observed in others (Keysers et al., 2018). This is supported by studies showing that electrical stimulation of the premotor cortex affects movements rather than conscious sensations, whereas electrostimulation in SI and the inferior parietal lobe results in conscious experiences and sensations (Desmurget & Sirigu, 2015).

A major aim of our study was to examine neural correlates of touch observation beyond SI and SII. Our results show several clusters that are commonly activated during touch observation: The first cluster includes the postcentral gyrus as well as the superior temporal gyrus (STG) and extends to the posterior insula. This is in line with the assumption that our own somatosensory network is vicariously activated when we see someone else being touched (e.g., Blakemore et al., 2005) and may be related to empathic feelings. Although imaging studies examining empathy usually emphasize the role of the anterior insula (Singer et al., 2004), previous research also suggested a role for somatosensory cortices in this respect (e.g., Banissy et al., 2012; Lamm et al., 2011; Peled-Avron et al., 2016). The posterior insula has been related to altered states of bodily awareness (Craig, 2009; Kuehn et al., 2016) but also to observed affective touch (Morrison et al., 2011). Nevertheless, the exact role of the posterior insula for empathy remains to be clarified. The STG region is well-known to represent a hub for a network of social perception (Isik et al., 2017) and is thought to play a crucial role for the evolution of social skills in humans (Patel et al., 2019).

While our results demonstrate an involvement of subcortical structures such as the thalamus in many MACM clusters, we did not find the medial temporal lobe to have co-activations with the ALE clusters of touch observation (e.g., BAs 28, 34-36) (see results of MACM). Whereas previous studies report that the observer’s past experiences can trigger the engagement of the action observation network (Calvo-Merino et al., 2005, 2006), and whereas memory networks are involved in the ad hoc experiences of pain and touch (see for review Gentsch & Kuehn, 2022), the medial temporal lobe and associated episodic memory networks may be less involved during observing touch of other people.

However, if not supported by episodic memory, how do we then understand seen touch? One answer may be that we process observed touch experiences by mental imagery together with perspective taking rather than by the activation of memory traces. Thus, we may be capable of imagining how tactile perceptions in others may feel without thinking of past episodic experiences of being touched in a similar way. This is supported by recent research demonstrating that mental imagery of touch (on our own body) recruits the somatosensory cortex in a topographic way (Schmidt & Blankenburg, 2019). These processes independent of episodic memory also support the view of a highly social brain (Burnett et al., 2011). However, alternative explanations may point to the recruitment of memory traces that are not based on medial temporal lobe activations, for example, because they are highly overlearned, they are not represented as a declarative memory and/or involve procedural memory sources. In addition, it is worth noting that medial temporal lobe structures involved in memory, such as subfields of the hippocampus but also the perirhinal cortex extending to the entorhinal cortex (Bonda et al., 1996), are small, have often low signal-to-noise-ratios in fMRI experiments, and may sometimes be missed using standard imaging approaches particularly when data are averaged and smoothed. Future analyses are therefore required to support this finding.

Another often posed question is: When observed and experienced touch both engage the somatosensory cortices, how does the brain differentiate between both? A first hypothesis claims that different portions of the somatosensory cortex mediate this crucial distinction. For example, BA2 mediates both physically perceived and observed touch whereas BA3b only mediates physically perceived touch (Bastiaansen et al., 2009; Keysers et al., 2010). However, a previous study using 7 T fMRI demonstrated that fine-grained topographic maps in BA3b are activated during touch observation (e.g., Kuehn et al., 2018). A second hypothesis on how the brain differentiates seen and experienced touch suggests a role for subcortical structures in this task. More precisely, it has been suggested that only physically experienced touch involves subcortical structures such as the thalamus (Keysers et al., 2010). Our finding of a systematic involvement of subcortical structures (thalamus) during touch observation revealed by the MACM analysis may speak against this argument. However, MACM is built on numerous different experiments and thus exclusively represents coactivation patterns that are not directly based on the comparison of actual touch versus observed touch. It may therefore be possible that the degree of thalamic involvement provides a critical dimension along which brain computations can distinguish between self-perceived and observed touch. Nevertheless, the MACM results question the conclusion that thalamic involvement as such is the critical distinguishing factor between physically perceived and observed touch. As a third hypothesis, also the insula may play a crucial role in distinguishing observed from experienced touch (Farrer et al., 2003; Karnath et al., 2005). Research here seems not conclusive. For example, Ebisch et al. reported that the posterior insula does not represent vicarious touch seen in others (Ebisch et al., 2011). This is supported by Heydrich and Blanke, who reported illusory own-body feelings subsequent lesions in the posterior insula (Heydrich & Blanke, 2013), suggesting that this brain region may be important for experienced rather than observed touch. In contrast to these studies, Morrison et al. demonstrated an involvement of the posterior insula when seeing touch (Morrison et al., 2011). Similar to Morrison et al., the present results do not support the idea that the insula may be crucial to differentiate self-experienced relative to mere observed touch. A fourth hypothesis is that, similar to the thalamus, it is mainly the strength of the activation that distinguishes observed from perceived touch (Kuehn et al., 2018). A meta-analysis that systematically compared signal strength of the above outlined network between physically perceived and observed touch may help testing for this hypothesis specifically. In addition, to target this question, it would be beneficial to design experiments in which the physically perceived tactile cue and the observed tactile cue are exactly identical.

A further interesting result of our analyses is that the convergence clusters indicating activations by vicarious touch consistently show coactivations with the medial frontal cortex. MACM analysis revealed that the dorso-medial frontal lobe is linked to all of the 8 ALE clusters. Thus, higher-order control areas seem to trigger or regulate other nodes of the touch observation network and may differentiate actual from observed touch. Task-dependent or attention-related top-down modulation of somatosensory cortices have been demonstrated before (Braun et al., 2002; Schaefer et al., 2005). For example, it has been shown that meditation (paying attention to one’s own hand) resulted in improved tactile acuity of this body portion (Philipp et al., 2015). It has also been suggested that the activation of the somatosensory cortices enhances frontal brain activation during actual touch (e.g., attention; Schirmer et al., 2011), a mechanism that could also mediate this medial frontal cortical activation in the present study, and subsequent network modification. Finally, also an involvement of the medial frontal cortex in memory mechanisms during mental simulation has been suggested (Kaplan et al., 2017).

This view is supplemented by the finding that the fusiform gyrus (BA 37) is connected to the identified clusters. The fusiform face area is part of the ventral stream of the visual system and known to be involved in facial recognition (Kanwisher et al., 1997). A link from vicarious touch observation to the fusiform face area suggests that we may be able to rapidly recognize faces as well as seen touch and link them together. This view is also supported by a recent study that revealed anatomical pathways connecting the fusiform gyrus with the intraparietal sulcus (Jitsuishi & Yamaguchi, 2020).

Interestingly, the network of convergence clusters resulting from the MACM analysis is very similar to the dorsal attention network, which includes the dorsal lateral prefrontal cortex, the frontal eye field, and the superior parietal lobe (intraparietal sulcus). This bilateral large-scale network is known to play a role in orienting attention, it holds the attention to focus on a cognitive task (Szczepanski et al., 2013). The network seems to represent the voluntary orienting of visuospatial attention towards the stimuli (the touch) the participants are observing. The dorsal attention network has been linked to top-down attention processes, which have been shown to improve and develop from early childhood to adolescence (Jolles et al., 2011).

What are the implications of these findings for our understanding of how the human brain processes the observation of touch? Above, we pointed out that our results (and the results of other studies, e.g., Schirmer et al., 2011) may refer to bottom-up processes, enabling us to rapidly recognize and evaluate a social situation. Touch observation may include both bottom-up and top-down processes, which might implicate that although touch observation may work in an automatic and unconscious way (bottom-up), it also has conscious parts that can be trained and that regulate the network. This may also be reflected by the results of the behavioral characterization and MACM results, which demonstrated connections predominantly to domains and networks related to action, cognition, and perception. The interaction between somatosensory and medial frontal networks may mediate the steady attention towards an observed tactile event, whereas evidence for an interaction between the medial frontal cortex and the hippocampus could not be found in this study.

The resulting clusters of the ALE-analysis were not equally distributed across the two hemispheres. This asymmetry might be caused by studies in which participants observed only one body part (e.g., the right hand) instead of both a right and left body part (e.g., Keysers et al., 2004). An alternative explanation refers to the hemispheric functional asymmetry for the social brain, which has been shown even for animals (Daisley et al., 2009) and also includes somatosensory brain regions. For example, Ruby and Decety (2004) reported that empathy in complex situations is linked to an activation of the right SI.

Some limitations of our study have to be mentioned. A total of 17-20 experiments is recommended for meaningful ALE results, but only 15 experiments were included in the current study (Müller et al., 2018). Thus, there is a risk that the results are driven by a few dominant studies and small effects could not be detected. However, calculation of the FSN modified for ALE shows that clusters C1–C6 fall between the upper and lower boundaries of the FSN, suggesting that there are no dominant studies influencing the results and also indicating high robustness of the data (Acar et al., 2018). The clusters C7 and C8 are below the lower threshold and should be considered preliminary until confirmed by future meta-analyses with more experiments.

Further limitations are more general and common to all ALE studies. A first point refers to the studies included in our analysis. Based on our approach, we did not include studies based on ROI analyses. Therefore, many studies on touch observation could not be considered. A second point touches the differences of the paradigms in our studies. ALE is a coordinate-based meta-analytic approach, in which each included study is represented by a set of locations of peak activation. Thus, the included studies differ with respect to the paradigm and the task for the participants. For example, although all studies addressed neural correlates of observed touch, some studies compared observed touch to non-touch, whereas others examined touch to a human subject relative to an unanimous object. Related to this, in many studies, the participants solved a task (e.g., count the touching strokes), whereas other studies aimed to record vicarious touch without any concrete task. A third limitation refers to effect sizes, which are not used to weight the studies (in contrast to behavioral meta-analytical approaches). Finally, it is difficult to take publication bias (except the file drawer problem) or study quality into account. However, we tried to address these issues by using the most recent guidelines (Acar et al., 2018; Müller et al., 2018) for our procedures to make our results reliable. We therefore consider the results helpful in guiding further approaches to understand the processing of observed touch experiences.

In conclusion, our findings present for the first time a meta-analysis of the neural networks of touch observation. We show that observation of touch is not strongly linked to episodic memory-related networks in the medial temporal lobe, suggesting that basic understanding of others may not to a large extent depend on the activation of episodic memory traces. In addition, we show that all networks related to touch observation are linked to an activation of the frontal lobes, which points to monitoring functions of these areas when seeing others being touched. Further research is needed to understand the neural processes of tactile empathy, a fundamental part of social perception.

## Supplementary Material

Supplementary Material

MACM_overlap_7a_axial

MACM_overlap_7b_coronal

MACM_overlap_7c_sagittal

Supplementary_FiguresS6a-h

## Data Availability

This meta-analysis used data provided by the authors of the original studies or taken from the original publications. All sources of these data can be found in the publication and/or in the supplementary material. The Foki files containing the extracted coordinates are available from the corresponding author upon reasonable request.
