# Supplementary material for: The neural networks of touch observation
Source: Imaging Neurosci (Camb). 2024 Jan 11;2:imag-2-00065. doi: 10.1162/imag_a_00065 (PMC12284895; doi:10.1162/imag_a_00065)
Supplement: Supplementary Material [file imag_a_00065-supp.pdf]

Supplementary Material

The neural networks of touch observation

Michael Schaefer[1], Esther Kuehn[2,3,4], Felix Schweitzer[1], Markus Muehlhan[5,6]

1 Department of Psychology, Medical School Berlin, 12247 Berlin, Germany

2 Hertie Institute for Clinical Brain Research, 72076 Tübingen, Germany

3 Institute for Cognitive Neurology and Dementia Research (IKND), Otto-von-Guericke University Magdeburg, 39120 Magdeburg, Germany

4 DZNE Tübingen, 72076 Tübingen, Germany

5 Department of Psychology, Faculty of Human Sciences, Medical School Hamburg, 20457 Hamburg, Germany

6 ICAN Institute for Cognitive and Affective Neuroscience, Medical School Hamburg, 20457 Hamburg, Germany

Supplementary material:

Figure S1: Flow diagram on the different stages of the systematic literature search according to Page et al. (2021).

Figure S2: Foci distribution of experiments integrated in the meta-analysis.

Figure S3: Results of the ALE meta-analysis.

Figure S4: Paradigm Analysis of the ALE derived clusters.

Figure S5: Results of the additional ROI-to-ROI resting-state analysis.

Figure S6a-h: Web pages of 3D-representation of MACM results.

Video S7a-c: Videos showing axial, coronal, and sagittal views of MACM results.

Table S1: Demographic data and details of the included studies.

Table S2: Behavioral and Paradigm Analyses of the single clusters.

Table S3: MACM results for each of the 8 ALE-derived network-cluster.

Table S4: Checklist for neuroimaging meta-analysis according to Müller et al. (2018).

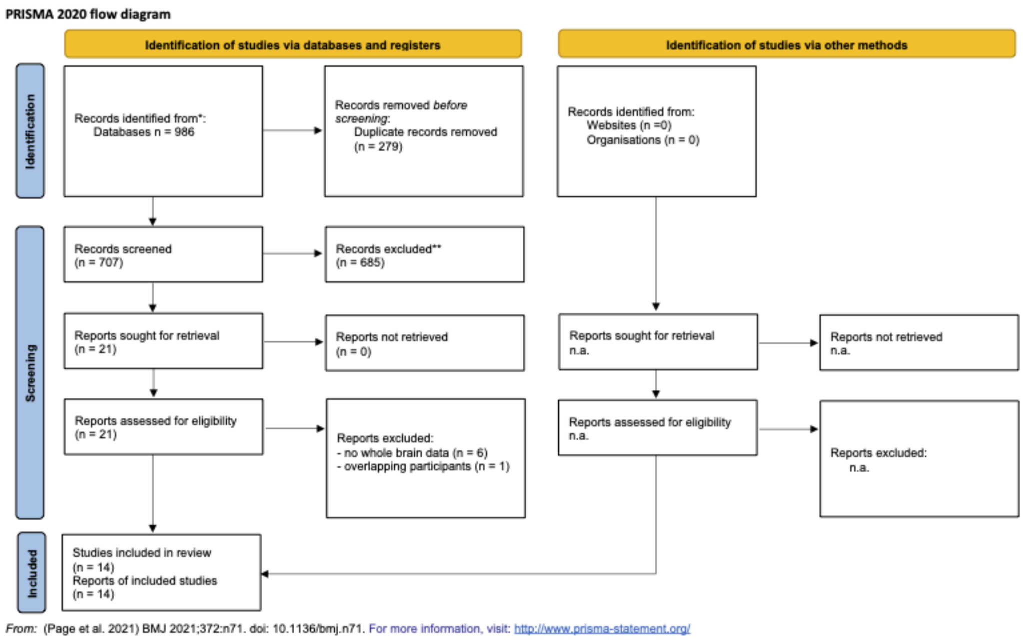

Figure S1. Flow diagram on the different stages of the systematic literature search according to Page et al. (2021).

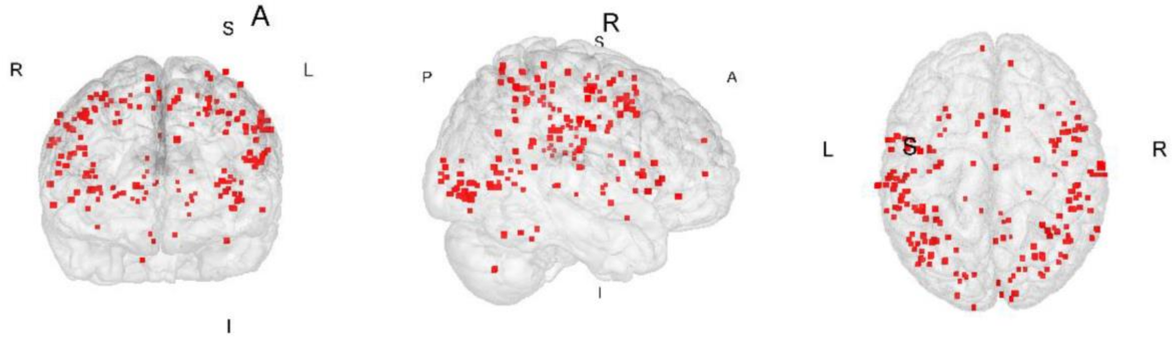

Figure S2: Foci distribution of experiments integrated in the meta-analysis. Foci are shown on a grey matter MNI glass brain. Red rectangles: Foci, A: anterior; S: superior; P: posterior; L: left; R: right. This image was created with Mango v4.1. (http://ric.uthsc sa.edu/mango /)

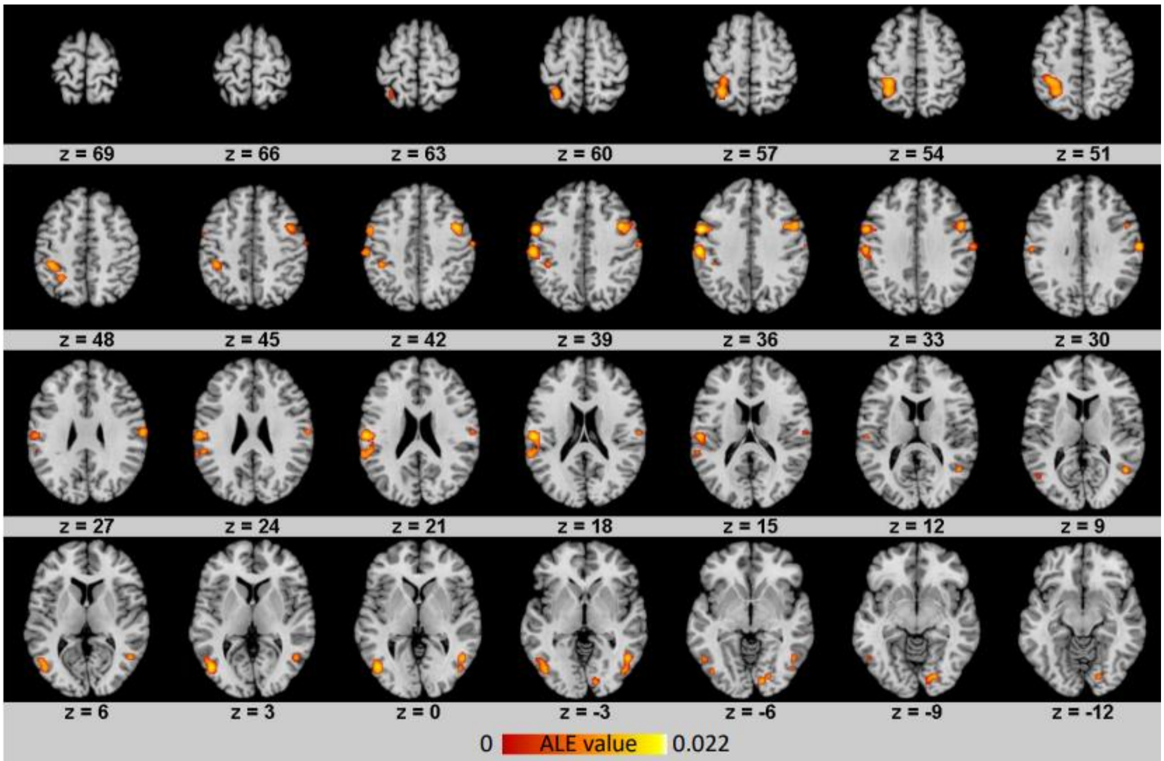

Figure S3: Results of the ALE meta-analysis. The clusters are depicted on axial slices of a MNI standard template in neurological convention. See Figure 1 in the main manuscript for a detailed identification of the clusters.

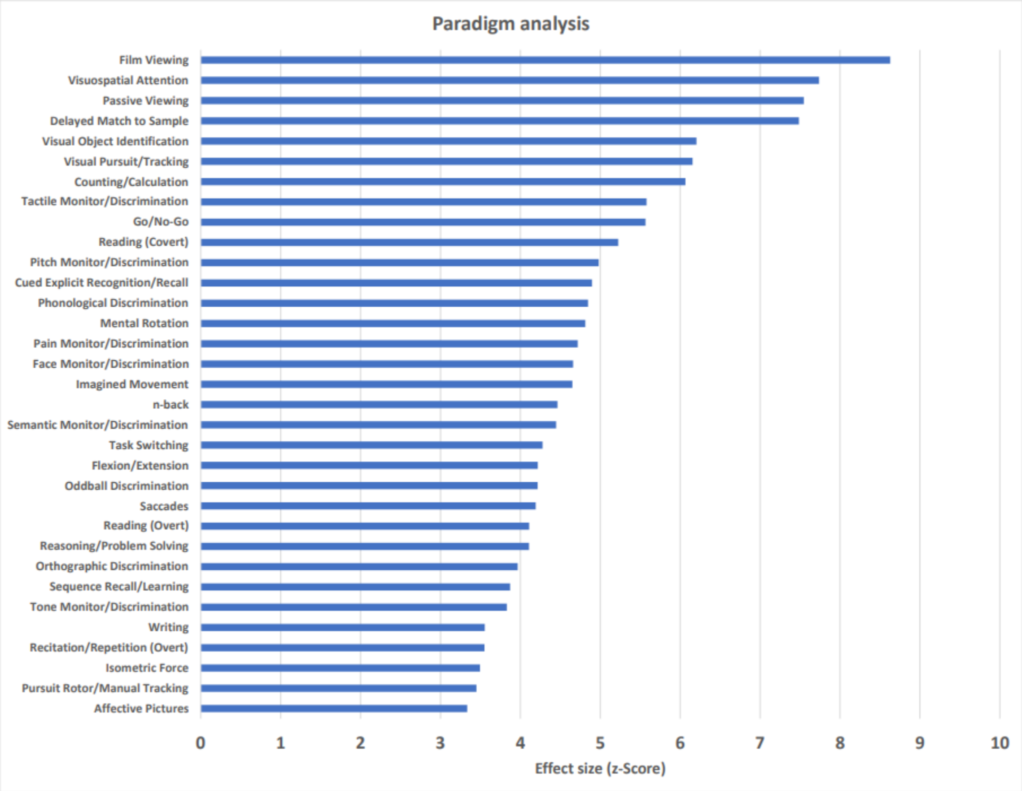

Figure S4: Paradigm Analysis of the ALE derived clusters. Only domains with an effect size of z > 3.3 are shown, as this is significant after correction for the size of the ROI/mask and the number of domains.

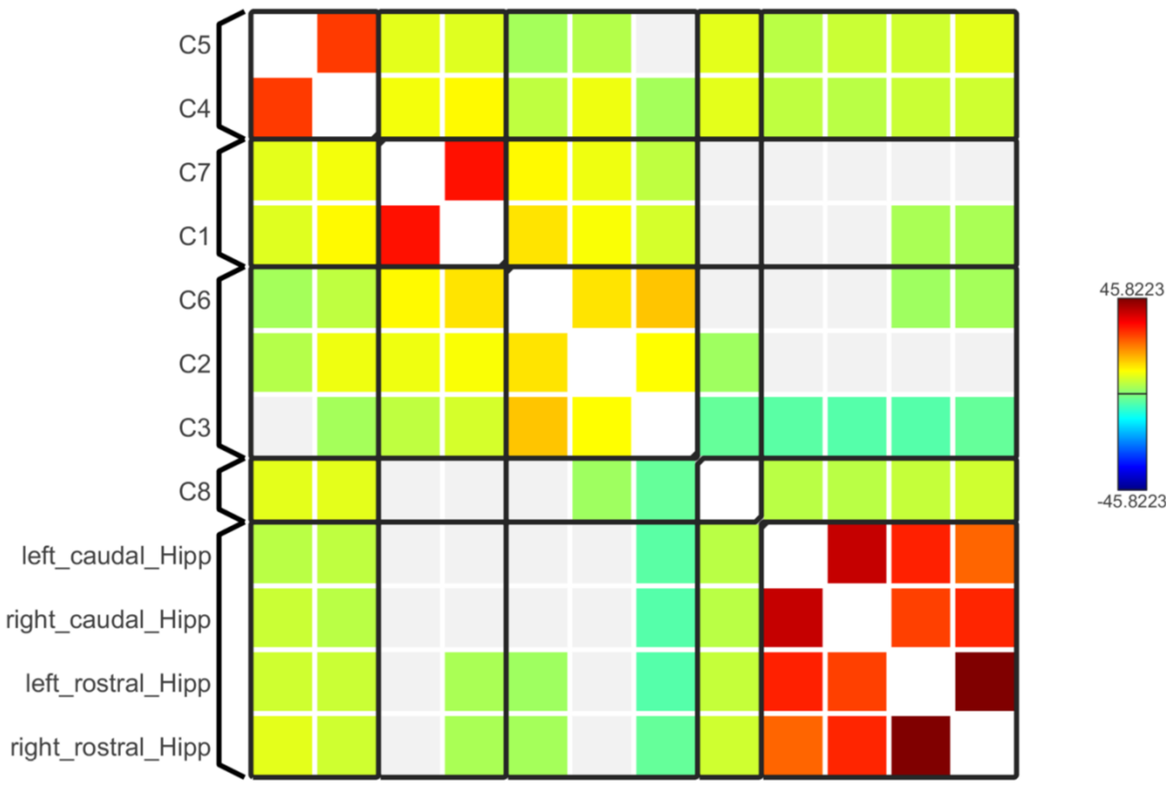

Figure S5: Results of the additional ROI-to-ROI resting-state analysis. Colours represent T-values from the second level of the General Linear Model ROI-to-ROI comparison, indicating the average deviation of connectivity from zero. Coloured fields indicate significant associations after correction for multiple comparisons.

Table S1

Characteristics of the studies included in the ALE-meta-analysis.

#	Sample Size (females)	Age, M (SD)	Template	Task	Control condition	Real touch	Body part	contrasts	Masked or whole brain	Task for participant	Design block or event
Keysers et al. (2004)	14 (6)	22-28 years	MNI	observe touched legs (by rod or brush)	observing same stimuli without touch	legs	legs	Vision of touch > vision of no-touch	Whole brain	No task	block
Blakemore, Bristow, Bird, Frith, and Ward (2005)	12	28.75 ±2.66	MNI	Observe neck of face touched by finger	Observe inanimate objects touched by finger	Neck and face	Neck and face	Vision of touch to human > vision of touch to object	Whole brain	No task	block
Ebisch et al. (2008)	15 (7)	19-27	Talairach	Observing intentional and accidental touch to humans and objects	Baseline	Brushing of hands	hands	Vison of touch > baseline	Whole brain	Count strokes	event-related
Schaefer, Xu, Flor, and Cohen (2009)	10 (7)	27 ±1.75	MNI	Observing touch to a hand using a paintbrush	observing same stimuli without touch	Brushing hands	hands	Vision of touch > vision of no-touch	Whole brain	Count strokes	block
Ebisch et al. (2011)	19 (10)	22-34	Talairach	Observing neutral or affective inanimate or skin-to-skin touch	baseline	Brushing hands with washing glove	hands	Vison of touch > baseline (fixation)	Whole brain	Count strokes	event-related
Cardini et al. (2011)	15 (10)	23.6	MNI	Observing touch to face or inanimate object	observing same stimuli without touch	Touch to face	face	Vision of touch > baseline (fixation)	Whole brain	Press butoon when feeling only one stroke	event-related

Schaefer, Heinze, and Rotte (2012a)	12 (6)	26	MNI	Observing touch to a hand using a paintbrush	observing same stimuli without touch	Touch to hand	hand	Vision of touch > vision of no-touch	masked	Count strokes	block
Schaefer, Heinze and Rotte (2012a, second study)	14 (7)	23	MNI	Observing touch to a hand using a paintbrush	observing same stimuli without touch	Touch to hand	hand	Vision of touch > vision of no-touch	masked	Count strokes	block
Schaefer, Heinze, and Rotte (2012b)	22 (11)	26	MNI	Observing touch to a hand using a paintbrush	observing same stimuli without touch	Touch to hand	hand	Vision of touch > vision of no-touch	masked	Count strokes	block
Meyer, Kaplan, Essex, Damasio, and Damasio (2011)	9 (4)	-	MNI	Observing a hand engaged in haptic exploration	Baseline (fixation)	-	hand	Vision of touch > baseline (fixation)	Whole brain	Watch videos	block
Holle, Banissy, and Ward (2013)	16 (11)	20-43	MNI	Observing touch to a face or object using a paintbrush	observing same stimuli without touch	Touch to face	face	Vision of touch > vision of no-touch	masked	Count strokes	block
Lee Masson, Van De Plas, Daniels, and Op de Beeck (2018))	22 (10)	26	MNI	Observing interpersonal touch	Observing non social touch	Touch to forearm	body	Vision of social touch > vision of non-social touch	Whole brain	Press button when agent has specific colored cloth	block
Kim, Bülthoff, Kim, and Bülthoff (2019)	15 (9)	26.7 ±3.6	MNI	Observing tactile explorations of a hand	baseline	Touch to hand	hand	Vision of touch > baseline	Whole brain	No task	block
Lee Masson et al. (2019))	21 (21)	23.9 ±2.8	MNI	Observing interpersonal touch	Observing non social touch	Touch to forearm	body	Vision of social touch > vision of non-social touch	Whole brain	Press button when agent has specific colored cloth	block
Ionta et al. (2020)	14 (8)	23.6 ±2.5	MNI	Observing touch to a human, animal, or robotic limb	Observing static limb	-	hand	Vision of touch > vision of static hand	Whole brain	Attend to video	block

Blakemore, S. J., Bristow, D., Bird, G., Frith, C., et al. (2005). Somatosensory activations during the observation of touch and a case of vision-touch synaesthesia. *Brain, 128*(Pt 7), 1571-1583.

Cardini, F., Costantini, M., Galati, G., Romani, G. L., et al. (2011). Viewing one's own face being touched modulates tactile perception: an fMRI study. *J Cogn Neurosci, 23*(3), 503-513.

Ebisch, S. J., Ferri, F., Salone, A., Perrucci, M. G., et al. (2011). Differential involvement of somatosensory and interoceptive cortices during the observation of affective touch. *J Cogn Neurosci, 23*(7), 1808-1822.

Ebisch, S. J., Perrucci, M. G., Ferretti, A., Del Gratta, C., et al. (2008). The sense of touch: embodied simulation in a visuotactile mirroring mechanism for observed animate or inanimate touch. *J Cogn Neurosci, 20*(9), 1611-1623.

Holle, H., Banissy, M. J., & Ward, J. (2013). Functional and structural brain differences associated with mirror-touch synaesthesia. *Neuroimage, 83*, 1041-1050.

Ionta, S., Costantini, M., Ferretti, A., Galati, G., et al. (2020). Visual similarity and psychological closeness are neurally dissociable in the brain response to vicarious pain. *Cortex, 133*, 295-308.

Keysers, C., Wicker, B., Gazzola, V., Anton, J. L., et al. (2004). A touching sight: SII/PV activation during the observation and experience of touch. *Neuron, 42*(2), 335-346.

Kim, J., Bülthoff, I., Kim, S. P., & Bülthoff, H. H. (2019). Shared neural representations of tactile roughness intensities by somatosensation and touch observation using an associative learning method. *Sci Rep, 9*(1), 77.

Lee Masson, H., Pillet, I., Amelynck, S., Van De Plas, S., et al. (2019). Intact neural representations of affective meaning of touch but lack of embodied resonance in autism: a multi-voxel pattern analysis study. *Mol Autism, 10*, 39.

Lee Masson, H., Van De Plas, S., Daniels, N., & Op de Beeck, H. (2018). The multidimensional representational space of observed socio-affective touch experiences. *Neuroimage, 175*, 297-314.

Meyer, K., Kaplan, J. T., Essex, R., Damasio, H., et al. (2011). Seeing touch is correlated with content-specific activity in primary somatosensory cortex. *Cereb Cortex, 21*(9), 2113-2121.

Schaefer, M., Heinze, H. J., & Rotte, M. (2012a). Close to you: embodied simulation for peripersonal space in primary somatosensory cortex. *PLoS One, 7*(8), e42308.

Schaefer, M., Heinze, H. J., & Rotte, M. (2012b). Embodied empathy for tactile events: Interindividual differences and vicarious somatosensory responses during touch observation. *Neuroimage, 60*(2), 952-957.

Schaefer, M., Xu, B., Flor, H., & Cohen, L. G. (2009). Effects of different viewing perspectives on somatosensory activations during observation of touch. *Hum Brain Mapp, 30*(9), 2722-2730.

Table S2
Behavioral and Paradigm Analyses of the single clusters

Cluster #	Behavioral Analyses Domain (- Category)	Z-Score[a]	Paradigm Analyses Paradigm Class	Z-Score[b]
1	Action		Pain Monitor/Discrimination	6.15
	- Execution (Unspecified)	6.76	Finger Tapping/Button Press	4.73
	Cognition		Pitch Monitor/Discrimination	4.48
	- Language (Speech)	4.34	Reading (Overt)	3.55
	- Music	3.59	Tone Monitor/Discrimination	3.48
	Interoception		Acupuncture	3.46
	- Sexuality	3.05	Tactile Monitor/Discrimination	3.40
	Perception		Oddball Discrimination	3.32
	- Audition	6.55		
	- Somesthesis (Pain)	5.85		
	- Somesthesis (Unspecified)	5.42		
2	Action		Finger Tapping/Button Press	6.77
	- Execution (Unspecified)	7.04	Visuospatial Attention	5.42
	- Observation	4.06	Delayed Match to Sample	4.66
	Cognition		Saccades	4.49
	- Attention	7.82	Visual Object Identification	4.19
	- Memory (Working)	6.33	Cued Explicit Recognition/Recall	4.17
	- Reasoning	5.66	Visual Pursuit/Tracking	4.01
	- Memory (Expliicit)	4.31	Task Switching	3.92
	- Language (Speech)	3.89	Counting/Calculation	3.91
	- Language (Semantics)	3.78	n-back	3.88
	- Spatial	3.35	Orthographic Discrimination	3.64
	- Language (Orthography)	3.19	Film Viewing	3.50
	Perception		Tactile Monitor/Discrimination	3.39
	- Vision (Motion)	6.76		
	- Vision (Unspecified)	4.34		
	- Vision (Shape)	4.08		
3	Action			
	- Execution (Unspecified)	4.12	Go/No-Go	4.36
	- Inhibition	4.05	Counting/Calculation	4.13
	Cognition		Visuospatial Attention	3.98
	- Attention	6.60	Finger Tapping/Button Press	3.94
	- Memory (Working)	4.34	Delayed Match to Sample	3.44
	- Reasoning	3.85	Film Viewing	3.32
	- Spatial	3.24		
	Interoception			
	- Sexuality	4.11		
	Perception			
	- Vision (Unspecified)	4.25		
	- Vision (Shape)	3.55		
4	Action		Passive Viewing	6.14
	- Observation	5.43	Film Viewing	5.29
	Cognition		Delayed Match to Sample	4.04
	- Attention	4.87	Visual Pursuit/Tracking	3.95
	- Language (Speech)	3.56	Face Monitor/Discrimination	3.46
	- Memory (Working)	3.11	Visuospatial Attention	3.41
	Interoception			
	- Sexuality	4.56		
	Perception			
	- Vision (Motion)	4.51		
	- Vision (Unspecified)	4.36		
	- Vision (Shape)	4.36		

a the significance threshold was set at Z-Scores ≥ 3.0 with $p \leq 0.05$ (corrected for multiple comparisons).
b the significance threshold was set at Z-Scores ≥ 3.3 with $p \leq 0.05$ (corrected for multiple comparisons).
n. s.= no results with values above significance thresholds.

Table S2 continued

Behavioral and Paradigm Analyses of the single clusters

Cluster #	Behavioral Analyses<br>Domain (- Category)	Z-Score[a]	Paradigm Analyses<br>Paradigm Class	Z-Score[b]
5	Action		Passive Viewing	5.20
	- Observation	4.16	Film Viewing	4.12
	Cognition		Face Monitor/Discrimination	3.09
	- Attention	3.84		
	Interoception			
	- Sexuality	4.60		
	Perception			
	- Vision (Shape)	4.45		
6	Action		Finger Tapping/Button Press	4.61
	- Execution (Unspecified)	6.07	Delayed Match to Sample	4.25
	Cognition		Visuospatial Attention	3.68
	- Attention	4.54	Reading (Covert)	3.68
	- Memory (Working)	4.41		
	- Language (Semantics)	4.23		
	- Language (Speech)	4.00		
	- Reasoning	3.68		
	- Language (Orthography)	3.27		
	- Language (Phonology)	3.09		
	Perception			
	- Vision (Unspecified)	3.22		
7	n.s.		n.s	
8	n.s.		n.s	

a the significance threshold was set at Z-Scores ≥ 3.0 with $p \leq 0.05$ (corrected for multiple comparisons).
b the significance threshold was set at Z-Scores ≥ 3.3 with $p \leq 0.05$ (corrected for multiple comparisons).
n. s.= no results with values above significance thresholds.

Table S3a

MACM Results Cluster 1. All results were significant at a cluster-forming threshold of $p < 0.001$ and cluster level FWE corrected at $p < 0.05$.

# Sub-Cluster	x	y	z	Cluster size (mm³)	Label
1	-56	-24	36	178936	Left Cerebrum.Parietal Lobe.Inferior Parietal Lobule.Gray Matter.Brodmann area 40
1	-12	-16	4		Left Cerebrum.Sub-lobar.Thalamus.Gray Matter.*
1	12	-14	6		Right Cerebrum.Sub-lobar.Thalamus.Gray Matter.Medial Dorsal Nucleus
1	-54	6	22		Left Cerebrum.Frontal Lobe.Inferior Frontal Gyrus.Gray Matter.Brodmann area 9
1	-52	6	2		Left Cerebrum.Frontal-Temporal Space.*.*.*
1	-38	-22	58		Left Cerebrum.Parietal Lobe.Postcentral Gyrus.*.*
1	-22	-2	0		Left Cerebrum.Sub-lobar.Lentiform Nucleus.Gray Matter.Putamen
1	22	4	4		Right Cerebrum.Sub-lobar.Lentiform Nucleus.Gray Matter.Putamen
1	-38	-42	50		Left Cerebrum.Parietal Lobe.Inferior Parietal Lobule.Gray Matter.Brodmann area 40
1	20	-2	0		Right Cerebrum.Sub-lobar.Lentiform Nucleus.Gray Matter.Lateral Globus Pallidus
1	-24	-64	52		Left Cerebrum.Parietal Lobe.Superior Parietal Lobule.Gray Matter.Brodmann area 7
1	-50	-4	46		Left Cerebrum.Frontal Lobe.Precentral Gyrus.Gray Matter.Brodmann area 4
1	46	-2	46		Right Cerebrum.Frontal Lobe.Precentral Gyrus.Gray Matter.Brodmann area 6
1	-48	-12	48		Left Cerebrum.Frontal Lobe.Precentral Gyrus.Gray Matter.Brodmann area 4
1	-40	-18	14		Left Cerebrum.Sub-lobar.Insula.Gray Matter.Brodmann area 13
1	44	-2	56		Right Cerebrum.Frontal Lobe.Precentral Gyrus.Gray Matter.Brodmann area 6
1	-28	-4	56		Left Cerebrum.Frontal Lobe.Middle Frontal Gyrus.Gray Matter.Brodmann area 6
1	14	-18	-10		Right Brainstem.Midbrain.*.Gray Matter.Subthalamic Nucleus
1	-22	-6	-16		Left Cerebrum.Limbic Lobe.Parahippocampal Gyrus.Gray Matter.Amygdala
2	0	0	58	23824	Left Cerebrum.Frontal Lobe.Medial Frontal Gyrus.*.*
2	2	12	38		Inter-Hemispheric.*.*.*.*
2	0	10	42		Left Cerebrum.Limbic Lobe.Cingulate Gyrus.*.*

MACM analysis is based on 353 experiments with 5953 foci and 4974 participants; specifications are limited to grey matter labels; *undefined sub-labels

Table S3b

MACM Results Cluster 2. All results were significant at a cluster-forming threshold of $p < 0.001$ and cluster level FWE corrected at $p < 0.05$.

# Sub-Cluster	x	y	z	Cluster size (mm³)	Label
1	-48	6	30	326088	Left Cerebrum.Frontal Lobe.Inferior Frontal Gyrus.Gray Matter.Brodmann area 6
1	32	-54	50		Right Cerebrum.Parietal Lobe.Superior Parietal Lobule.Gray Matter.Brodmann area 7
1	-2	8	52		Left Cerebrum.Frontal Lobe.Medial Frontal Gyrus.Gray Matter.Brodmann area 6
1	0	18	48		Left Cerebrum.Frontal Lobe.Superior Frontal Gyrus.Gray Matter.Brodmann area 6
1	-26	-4	56		Left Cerebrum.Frontal Lobe.Middle Frontal Gyrus.Gray Matter.Brodmann area 6
1	40	0	50		Right Cerebrum.Frontal Lobe.Precentral Gyrus.*.*
1	-12	-16	6		Left Cerebrum.Sub-lobar.Thalamus.Gray Matter.*
1	44	-54	-18		Right Cerebrum.Temporal Lobe.Fusiform Gyrus.Gray Matter.Brodmann area 37
1	12	-12	6		Right Cerebrum.Sub-lobar.Thalamus.Gray Matter.*
1	32	-90	2		Right Cerebrum.Occipital Lobe.Middle Occipital Gyrus.Gray Matter.*
1	-20	6	2		Left Cerebrum.Sub-lobar.Lentiform Nucleus.Gray Matter.Putamen
1	36	-64	-24		Right Cerebellum.Posterior Lobe.Declive.Gray Matter.*
1	20	8	2		Right Cerebrum.Sub-lobar.Lentiform Nucleus.Gray Matter.Putamen
1	-12	0	12		Left Cerebrum.Sub-lobar.Caudate.Gray Matter.Caudate Body
1	-50	10	0		Left Cerebrum.Sub-lobar.Insula.*.*
1	18	-96	4		Right Cerebrum.Occipital Lobe.Lingual Gyrus.Gray Matter.Brodmann area 17
1	-60	-20	28		Left Cerebrum.Parietal Lobe.Inferior Parietal Lobule.Gray Matter.Brodmann area 40
1	-8	-20	-8		Left Brainstem.Midbrain.*.Gray Matter.Red Nucleus
1	62	-20	36		Right Cerebrum.Parietal Lobe.Postcentral Gyrus.Gray Matter.Brodmann area 2
1	62	-32	20		Right Cerebrum.Sub-lobar.Insula.Gray Matter.Brodmann area 13
1	60	-24	20		Right Cerebrum.Parietal Lobe.Postcentral Gyrus.Gray Matter.Brodmann area 40
1	-54	-24	18		Left Cerebrum.Parietal Lobe.Postcentral Gyrus.Gray Matter.Brodmann area 40
1	22	-30	-4		Right Brainstem.Midbrain.*.*.*

MACM analysis is based on 948 experiments with 15313 foci and 14766 participants; specifications are limited to grey matter labels; *undefined sub-labels

Table S3c

MACM Results Cluster 3. All results were significant at a cluster-forming threshold of $p < 0.001$ and cluster level FWE corrected at $p < 0.05$.

# Sub-Cluster	x	y	z	Cluster size (mm³)	Label
1	50	8	30	172680	Right Cerebrum.Frontal Lobe.Inferior Frontal Gyrus.Gray Matter.Brodmann area 9
1	-46	6	30		Left Cerebrum.Frontal Lobe.Inferior Frontal Gyrus.Gray Matter.Brodmann area 6
1	0	10	50		Left Cerebrum.Frontal Lobe.Superior Frontal Gyrus.*.*
1	2	16	46		Left Cerebrum.Frontal Lobe.Medial Frontal Gyrus.*.*
1	10	-16	8		Right Cerebrum.Sub-lobar.Thalamus.Gray Matter.Medial Dorsal Nucleus
1	-28	-4	52		Left Cerebrum.Frontal Lobe.Precentral Gyrus.Gray Matter.Brodmann area 6
1	-10	-16	6		Left Cerebrum.Sub-lobar.Thalamus.Gray Matter.Medial Dorsal Nucleus
1	32	-2	50		Right Cerebrum.Frontal Lobe.Middle Frontal Gyrus.*.*
1	22	6	2		Right Cerebrum.Sub-lobar.Lentiform Nucleus.Gray Matter.Putamen
1	-22	2	2		Left Cerebrum.Sub-lobar.Lentiform Nucleus.Gray Matter.Putamen
1	8	-22	-8		Right Brainstem.Midbrain.*.Gray Matter.Red Nucleus
1	-6	-24	-6		Left Brainstem.Midbrain.*.*.*
2	44	-62	-12	53688	Right Cerebrum.Temporal Lobe.Fusiform Gyrus.Gray Matter.Brodmann area 37
2	62	-30	24		Right Cerebrum.Parietal Lobe.Inferior Parietal Lobule.*.*
2	32	-62	-28		Right Cerebellum.Anterior Lobe.Culmen.Gray Matter.*
3	-54	-40	24	47312	Left Cerebrum.Temporal Lobe.Superior Temporal Gyrus.*.*

MACM analysis is based on 519 experiments with 9474 foci and 7990 participants; specifications are limited to grey matter labels; *undefined sub-labels

Table S3d

MACM Results Cluster 4. All results were significant at a cluster-forming threshold of $p < 0.001$ and cluster level FWE corrected at $p < 0.05$.

# Sub-Cluster	x	y	z	Cluster size (mm³)	Label
1	-48	6	28	94944	Left Cerebrum.Frontal Lobe.Precentral Gyrus.Gray Matter.Brodmann area 6
1	-42	-54	-18		Left Cerebrum.Temporal Lobe.Fusiform Gyrus.Gray Matter.Brodmann area 37
1	-20	-64	56		Left Cerebrum.Parietal Lobe.Precuneus.Gray Matter.Brodmann area 7
1	-46	0	44		Left Cerebrum.Frontal Lobe.Precentral Gyrus.Gray Matter.Brodmann area 6
1	-26	-6	54		Left Cerebrum.Frontal Lobe.Precentral Gyrus.Gray Matter.Brodmann area 6
1	-32	-86	4		Left Cerebrum.Occipital Lobe.Middle Occipital Gyrus.Gray Matter.Brodmann area 18
2	30	-58	54	59136	Right Cerebrum.Parietal Lobe.Superior Parietal Lobule.Gray Matter.Brodmann area 7
2	32	-90	2		Right Cerebrum.Occipital Lobe.Middle Occipital Gyrus.Gray Matter.*
2	44	-40	48		Right Cerebrum.Parietal Lobe.Inferior Parietal Lobule.Gray Matter.Brodmann area 40
2	20	-96	8		Right Cerebrum.Occipital Lobe.Cuneus.Gray Matter.Brodmann area 17
3	48	8	28	28280	Right Cerebrum.Frontal Lobe.Inferior Frontal Gyrus.Gray Matter.Brodmann area 9
3	30	-4	50		Right Cerebrum.Frontal Lobe.Middle Frontal Gyrus.Gray Matter.Brodmann area 6
3	48	2	44		Right Cerebrum.Frontal Lobe.Precentral Gyrus.Gray Matter.Brodmann area 6
4	-2	8	54	12520	Left Cerebrum.Frontal Lobe.Medial Frontal Gyrus.Gray Matter.Brodmann area 6
4	-2	28	34		Left Cerebrum.Frontal Lobe.Cingulate Gyrus.Gray Matter.Brodmann area 32
5	-12	-18	4	7320	Left Cerebrum.Sub-lobar.Thalamus.Gray Matter.Mammillary Body
5	10	-18	4		Right Cerebrum.Sub-lobar.Thalamus.Gray Matter.*

MACM analysis is based on 402 experiments with 6550 foci and 6216 participants; specifications are limited to grey matter labels; *undefined sub-labels

Table S3e

MACM Results Cluster 5. All results were significant at a cluster-forming threshold of $p < 0.001$ and cluster level FWE corrected at $p < 0.05$.

# Sub-Cluster	x	y	z	Cluster size (mm^3)	Label
1	44	-40	48	56864	Right Cerebrum.Parietal Lobe.Inferior Parietal Lobule.Gray Matter.Brodmann area 40
1	26	-86	-14		Right Cerebellum.Posterior Lobe.Declive.Gray Matter.*
1	18	-98	8		Right Cerebrum.Occipital Lobe.Cuneus.Gray Matter.Brodmann area 17
2	-28	-36	-18	30232	Left Cerebrum.Limbic Lobe.Parahippocampal Gyrus.*.*
3	50	8	26	22816	Right Cerebrum.Frontal Lobe.Inferior Frontal Gyrus.Gray Matter.Brodmann area 9
3	34	-4	50		Right Cerebrum.Frontal Lobe.Precentral Gyrus.Gray Matter.Brodmann area 6
3	56	20	12		Right Cerebrum.Frontal Lobe.Inferior Frontal Gyrus.Gray Matter.Brodmann area 44
4	-48	-32	40	22360	Left Cerebrum.Parietal Lobe.Inferior Parietal Lobule.Gray Matter.Brodmann area 40
5	-2	18	48	12712	Left Cerebrum.Frontal Lobe.Superior Frontal Gyrus.Gray Matter.Brodmann area 6
5	4	14	46		Right Cerebrum.Frontal Lobe.Medial Frontal Gyrus.*.*
5	-2	8	50		Left Cerebrum.Frontal Lobe.Medial Frontal Gyrus.Gray Matter.Brodmann area 6
5	0	8	56		Left Cerebrum.Frontal Lobe.Medial Frontal Gyrus.*.*
5	-2	22	26		Left Cerebrum.Limbic Lobe.Cingulate Gyrus.Gray Matter.Brodmann area 24
5	2	14	30		Left Cerebrum.Limbic Lobe.Cingulate Gyrus.*.*
5	-6	12	36		Left Cerebrum.Limbic Lobe.Cingulate Gyrus.Gray Matter.Brodmann area 24
6	-46	6	28	12664	Left Cerebrum.Frontal Lobe.Precentral Gyrus.*.*
6	-26	-4	50		Left Cerebrum.Frontal Lobe.Middle Frontal Gyrus.Gray Matter.Brodmann area 6
7	8	-16	8	8296	Right Cerebrum.Sub-lobar.Thalamus.Gray Matter.Medial Dorsal Nucleus
7	-10	-14	4		Left Cerebrum.Sub-lobar.Thalamus.Gray Matter.*
7	-6	-22	-10		Left Brainstem.Midbrain.*.*.*
7	-4	-28	-8		Left Brainstem.Midbrain.*.*.*
7	6	-26	-4		Right Brainstem.Midbrain.*.*.*

MACM analysis is based on 294 experiments with 4985 foci and 4868 participants; specifications are limited to grey matter labels; *undefined sub-labels

Table S3f

MACM Results Cluster 6. All results were significant at a cluster-forming threshold of $p < 0.001$ and cluster level FWE corrected at $p < 0.05$.

# Sub-Cluster	x	y	z	Cluster size (mm³)	Label
1	-2	8	52	98920	Left Cerebrum.Frontal Lobe.Medial Frontal Gyrus.Gray Matter.Brodmann area 6
1	50	8	28		Right Cerebrum.Frontal Lobe.Inferior Frontal Gyrus.Gray Matter.Brodmann area 9
1	-10	-18	6		Left Cerebrum.Sub-lobar.Thalamus.Gray Matter.Medial Dorsal Nucleus
1	10	-16	8		Right Cerebrum.Sub-lobar.Thalamus.Gray Matter.Medial Dorsal Nucleus
1	24	6	2		Right Cerebrum.Sub-lobar.Lentiform Nucleus.Gray Matter.Putamen
1	-24	-2	4		Left Cerebrum.Sub-lobar.Lentiform Nucleus.Gray Matter.Putamen
1	-12	-2	10		Left Cerebrum.Sub-lobar.Thalamus.Gray Matter.Ventral Anterior Nucleus
1	-6	-26	-8		Left Brainstem.Midbrain.*.*.*
2	-50	6	32	86936	Left Cerebrum.Frontal Lobe.Precentral Gyrus.*.*
2	-26	-4	54		Left Cerebrum.Frontal Lobe.Middle Frontal Gyrus.Gray Matter.Brodmann area 6
2	-52	12	0		Left Cerebrum.Frontal-Temporal Space.*.*.*
2	-60	-20	28		Left Cerebrum.Parietal Lobe.Inferior Parietal Lobule.Gray Matter.Brodmann area 40
2	-52	10	-12		Left Cerebrum.Temporal Lobe.Superior Temporal Gyrus.Gray Matter.Brodmann area 22
2	-54	-40	22		Left Cerebrum.Temporal Lobe.Superior Temporal Gyrus.*.*
2	-42	48	16		Left Cerebrum.Frontal Lobe.Middle Frontal Gyrus.Gray Matter.Brodmann area 10
3	32	-56	52	20384	Right Cerebrum.Parietal Lobe.Superior Parietal Lobule.Gray Matter.Brodmann area 7
4	-42	-70	-12	19328	Left Cerebrum.Occipital Lobe.Fusiform Gyrus.Gray Matter.Brodmann area 19
4	-36	-88	-6		Left Cerebrum.Occipital Lobe.Middle Occipital Gyrus.Gray Matter.Brodmann area 18
4	-28	-62	-24		Left Cerebellum.Anterior Lobe.Culmen.Gray Matter.*
4	-30	-94	10		Left Cerebrum.Occipital Lobe.Middle Occipital Gyrus.Gray Matter.Brodmann area 18
4	-16	-62	-20		Left Cerebellum.Anterior Lobe.*.Gray Matter.Dentate
5	32	-62	-26	11824	Right Cerebellum.Anterior Lobe.Culmen.Gray Matter.*
5	44	-54	-18		Right Cerebrum.Temporal Lobe.Fusiform Gyrus.Gray Matter.Brodmann area 37

MACM analysis is based on 416 experiments with 7117 foci and 6755 participants; specifications are limited to grey matter labels; *undefined sub-labels

Table S3g

MACM Results Cluster 7. All results were significant at a cluster-forming threshold of $p < 0.001$ and cluster level FWE corrected at $p < 0.05$.

# Sub-Cluster	x	y	z	Cluster size (mm³)	Label
1	-50	6	32	223456	Left Cerebrum.Frontal Lobe.Precentral Gyrus.*.*
1	-2	10	52		Left Cerebrum.Frontal Lobe.Medial Frontal Gyrus.*.*
1	50	8	28		Right Cerebrum.Frontal Lobe.Inferior Frontal Gyrus.Gray Matter.Brodmann area 9
1	6	16	44		Right Cerebrum.Frontal Lobe.Medial Frontal Gyrus.Gray Matter.Brodmann area 32
1	-10	-18	6		Left Cerebrum.Sub-lobar.Thalamus.Gray Matter.Medial Dorsal Nucleus
1	-26	-4	56		Left Cerebrum.Frontal Lobe.Middle Frontal Gyrus.Gray Matter.Brodmann area 6
1	12	-16	6		Right Cerebrum.Sub-lobar.Thalamus.Gray Matter.Medial Dorsal Nucleus
1	24	6	2		Right Cerebrum.Sub-lobar.Lentiform Nucleus.Gray Matter.Putamen
1	-24	-2	4		Left Cerebrum.Sub-lobar.Lentiform Nucleus.Gray Matter.Putamen
1	-54	10	0		Left Cerebrum.Frontal-Temporal Space.*.*.*
1	-14	-68	56		Left Cerebrum.Parietal Lobe.Precuneus.Gray Matter.Brodmann area 7
1	-62	-32	8		Left Cerebrum.Temporal Lobe.Superior Temporal Gyrus.Gray Matter.Brodmann area 42
1	60	-28	38		Right Cerebrum.Parietal Lobe.Inferior Parietal Lobule.Gray Matter.Brodmann area 40
1	-4	-24	-8		Left Brainstem.Midbrain.*.Gray Matter.Red Nucleus
1	10	-20	-10		Right Brainstem.Midbrain.*.Gray Matter.Red Nucleus
1	-54	-40	22		Left Cerebrum.Temporal Lobe.Superior Temporal Gyrus.*.*
2	-42	-70	-12	17560	Left Cerebrum.Occipital Lobe.Fusiform Gyrus.Gray Matter.Brodmann area 19
2	-28	-62	-24		Left Cerebellum.Anterior Lobe.Culmen.Gray Matter.*
2	-30	-94	10		Left Cerebrum.Occipital Lobe.Middle Occipital Gyrus.Gray Matter.Brodmann area 18
2	-14	-96	-4		Left Cerebrum.Occipital Lobe.Inferior Occipital Gyrus.Gray Matter.Brodmann area 17
3	28	-64	-24	11616	Right Cerebellum.Posterior Lobe.Declive.Gray Matter.*
3	44	-54	-18		Right Cerebrum.Temporal Lobe.Fusiform Gyrus.Gray Matter.Brodmann area 37
3	14	-56	-28		Right Cerebellum.Anterior Lobe.*.Gray Matter.Dentate

MACM analysis is based on 493 experiments with 7927 foci and 7868 participants; specifications are limited to grey matter labels; *undefined sub-labels

Table S3h

MACM Results Cluster 8. All results were significant at a cluster-forming threshold of $p < 0.001$ and cluster level FWE corrected at $p < 0.05$.

# Sub-Cluster	x	y	z	Cluster size (mm^3)	Label
1	22	-82	-12	21136	Right Cerebellum.Posterior Lobe.Declive.Gray Matter.*
1	20	-96	2		Right Cerebrum.Occipital Lobe.Lingual Gyrus.Gray Matter.Brodmann area 17
1	34	-90	4		Right Cerebrum.Occipital Lobe.Middle Occipital Gyrus.Gray Matter.Brodmann area 18
1	26	-58	-20		Right Cerebellum.Anterior Lobe.Culmen.Gray Matter.*
2	-16	-88	-12	18392	Left Cerebrum.Occipital Lobe.Lingual Gyrus.Gray Matter.Brodmann area 18
2	-34	-58	-16		Left Cerebellum.Posterior Lobe.Declive.Gray Matter.*
2	-22	-84	-16		Left Cerebellum.Posterior Lobe.Declive.Gray Matter.*
2	-10	-96	-6		Left Cerebrum.Occipital Lobe.Inferior Occipital Gyrus.Gray Matter.Brodmann area 17
3	-36	-44	52	7992	Left Cerebrum.Parietal Lobe.Inferior Parietal Lobule.Gray Matter.Brodmann area 40
3	-30	-54	56		Left Cerebrum.Parietal Lobe.Superior Parietal Lobule.Gray Matter.Brodmann area 7
4	-44	-2	50	7416	Left Cerebrum.Frontal Lobe.Precentral Gyrus.Gray Matter.Brodmann area 6
4	-34	-4	62		Left Cerebrum.Frontal Lobe.Precentral Gyrus.Gray Matter.Brodmann area 6
4	-26	2	60		Left Cerebrum.Frontal Lobe.Sub-Gyral.Gray Matter.Brodmann area 6
5	-2	6	54	5248	Left Cerebrum.Frontal Lobe.Medial Frontal Gyrus.Gray Matter.Brodmann area 6
5	-2	14	50		Left Cerebrum.Frontal Lobe.Superior Frontal Gyrus.Gray Matter.Brodmann area 6
6	32	-56	50	3176	Right Cerebrum.Parietal Lobe.Superior Parietal Lobule.Gray Matter.Brodmann area 7
7	54	10	36	2200	Right Cerebrum.Frontal Lobe.Middle Frontal Gyrus.Gray Matter.Brodmann area 9
7	50	8	34		Right Cerebrum.Frontal Lobe.Precentral Gyrus.Gray Matter.Brodmann area 6
8	38	-2	56	1768	Right Cerebrum.Frontal Lobe.Precentral Gyrus.Gray Matter.Brodmann area 6

MACM analysis is based on 77 experiments with 1272 foci and 1066 participants; specifications are limited to grey matter labels; *undefined sub-labels

Table S4

Checklist for neuroimaging meta-analysis according to Müller et al. (2018).

The research question is specifically defined	YES, and it includes the following contrast:<br><br>whole brain touch observation vs. whole brain control conditions<br><br>→ ***Introduction, last paragraph***
The literature search was systematic	YES, it included the following keywords in the following databases:<br><br>Keywords: (touch OR tactile) AND (observation OR sight OR vision) AND functional magnetic resonance imaging (fMRI).<br><br>Databases: PubMed, PsycINFO, Web of Science (up to September, 15th, 2021).<br><br>→ ***Methods section: Literature Search***
Detailed inclusion and exclusion criteria are included	YES, and reasons of non-standard criterion were:<br><br>Exclusion of studies using ROIs or restricting analyses to a slab of the brain in order to prevent biases towards those regions.<br><br>→ ***Methods section: Literature Search***
Sample overlap was taken into account	YES, using the following method:<br><br>Sample overlap was examined and authors were contacted if sample overlap was unclear.<br><br>→ ***Methods section: Literature Search***
All experiments use the same search coverage (state how brain coverage is assessed and how small volume corrections and conjunctions are taken into account)	YES, the search coverage is the following:<br><br>whole brain coverage only, verified via details of the scanner parameters provided in the method section of the papers<br><br>→ ***Methods section: Literature Search***
Studies are converted to a common reference space	Yes, using the following conversion:<br><br>Coordinates reported in Talairach space were transformed into MNI space using the Lancaster transform icbm2tal implemented in GingerALE.

	→ ***Methods section: Activation Likelihood Estimation***
Data extraction have been conducted by two investigators (ideal case) or double checked by the same investigator (state how double-checking was performed)	YES, the following authors:<br><br>FS checked the inclusion criteria<br>FS extracted coordinates<br>FS extracted other info: demographic sample characteristics and methodological characteristics<br>MS double-checked for the following data: all<br><br>→ ***Methods section: Activation Likelihood Estimation***
The paper includes a table with at least the references, basic study description (e. g. for fMRI tasks: stimuli), contrasts and basic sample descriptions (e.g. size, mean age and gender distribution, specific characteristics) of the included studies, source of information (e.g. contact with authors), reference space	YES, and also the following data:<br><br>Analysis: Control conditions, body part, tasks, design<br>Results: Reference Space<br><br>→ ***Table S1***
The study protocol was previously registered and all analyses planned beforehand, including the methods and parameters used for inference, correction for multiple testing, etc.	Yes:<br>1) The meta-analysis was registered before starting the starting the search at: PROSPERO (https://www.crd.york.ac.uk/prospero/display_record.php?RecordID=266316)<br><br>→ ***Methods section: Preregistration***<br><br>2) Any non-planned analyses are clearly stated as post-hoc or non-prespecified in the paper.<br>1. Connectivity of ALE clusters with task-independent resting-state analysis<br><br>→ ***Methods section, Resting State Analysis***<br><br>3) The meta-analysis used the default methods and parameters of the software.

	→ ***Methods section: Anatomical Likelihood Estimation***
The meta-analysis includes diagnostics	YES, the following:<br><br><u>Number of experiments contributing to the resulting ALE-clusters.</u><br><br>→ ***Table 1 and table S1***<br><br>Estimation of publication bias (file drawer problem) and the potential influence of dominant studies<br><br>→ ***Methods and results section***
